# Immobilization-driven enhancement of CNT-binding peptide affinity: substantial shift from the free-state

**DOI:** 10.1039/d5ra09758d

**Published:** 2026-05-08

**Authors:** Narangerel Ganbaatar, Huan-Wen Han, Naofumi Okamoto, Ting-Chieh Chu, Kenji Iwahori, Aghnia Dinan Maulani Heriyanto, Masakazu Nakamura, Ichiro Yamashita

**Affiliations:** a Graduate School of Engineering, The University of Osaka 2-8 Yamadaoka Suita Osaka 565-0871 Japan yamashita@ssc.eei.eng.osaka-u.ac.jp namynamy02@gmail.com; b Division of Materials Science, Nara Institute of Science and Technology Ikoma Nara 630-0192 Japan

## Abstract

Three carbon-binding peptide aptamers, Y1 (FKQDAWEAVDIR), Y2 (SYTHLLHRSLPG), and Y3 (SLHNAEMANFRA) were identified *via* phage display. Their adsorption efficiency was analyzed in the free state and immobilized on the cage-shaped protein Dps. Cyclic voltammetry (CV) showed that Y1 and Y2 had high affinities, while Y3 had lower affinity in its free state. Dps mutant proteins presenting Y1, Y2, and Y3 on the outer surface (Y1Ct, Y2Ct, Y3Ct) were then analyzed. Unexpectedly, Y3Ct exhibited a strong affinity for carbon surfaces. Quartz crystal microbalance (QCM) measurements, conducted in both batch and open-flow modes, quantified the adsorbed mass and dissociation constants (*K*_d_). Y3Ct had a much lower *K*_d_ (48 nM) than Y1Ct (1.04 µM) and Y2Ct (2.18 µM). These findings confirmed that aptamer adsorption efficiency depended on whether the aptamer was free or immobilized. Although the mechanism is unclear, a plausible explanation is that hydrophobic and electrostatic interactions near the C-terminus might have contributed to Y3Ct's enhanced affinity for the carbon surface.

## Abstract 2

Three CNT-binding peptide aptamers, Y1, Y2, and Y3, were selected by phage display. While Y3 showed low affinity in the free state, immobilization on a cage-shaped protein (Dps) dramatically enhanced its affinity, yielding a markedly lower *K*_d_ than Y1 and Y2.

## Graphical abstract

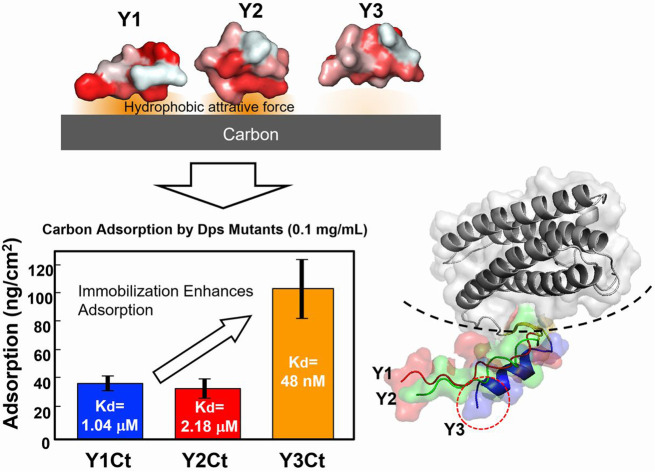

## Introduction

Ferritin, a cage-shaped protein ubiquitously distributed in living organisms, is a 24-mer iron storage protein in which excess Fe(ii) is oxidized at the catalytic sites of subunits and mineralized as iron oxide nanoparticles inside the cavity.^1,2^ Due to its biologically important function, ferritin has been extensively studied since the 1980s as a key interface linking inorganic materials with biomolecules. Its mineralization mechanisms and potential applications have been continuously explored.^1–5^

In the 1990s, engineering studies on nanoparticle synthesis of nanoparticles within the ferritin cavity advanced rapidly. Pioneering work by S. Mann, T. Douglas, I. Yamashita, and others reported the formation of magnetic, semiconductor, metal, and metal oxide nanoparticles.^6–11^ In parallel, A. M. Belcher and co-workers identified semiconductor surface-specific peptides by phage display,^12,13^ while K. Shiba and colleagues demonstrated selective binding to titanium and carbon nanohorns.^14,15^ S. Brown subsequently developed gold-binding peptides,^16^ and M. Sarikaya and co-workers identified a wide range of inorganic material-binding peptides.^17^ Collectively, these advances enabled the selective placement and organization of nanomaterials using peptide aptamers.

Based on these findings, it was proposed to functionalize ferritin containing a nanoparticle core with peptide aptamers for nanotechnological applications.^18–21^ As part of this broader effort, our group has focused on developing a method for nanoelectronic device fabrication using cage-shaped proteins, which we named the Bio Nano Process (BNP).^22^ We have demonstrated the self-organized fabrication of nanostructures in floating nanodot memory,^23^ resistive switching memory,^24^ dye-sensitized solar cells,^25^ and quantum optical devices.^26^

We are currently developing a CNT-based thermoelectric composite using DNA-binding protein from starved cells (Dps), a 12-mer small ferritin family protein with a molecular weight of approximately 240 kDa.^27^ Incorporating CNT/Dps(core)/CNT heterojunctions suppresses the high thermal conductivity of CNTs while preserving tunneling conduction, yielding nearly a three-order-of-magnitude improvement in the thermoelectric figure of merit (*ZT*).^27^ A central focus of this work is the development of CNT-binding peptides and Dps mutants with enhanced adsorption on CNTs or carbon surfaces.

**Fig. 1:**
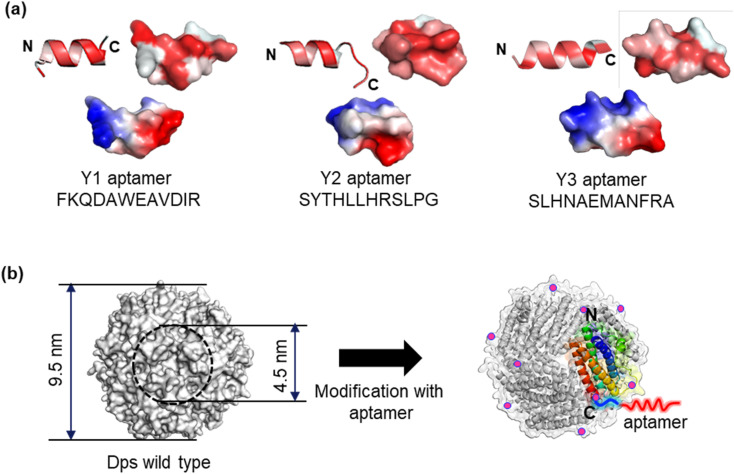
(a) Predicted models of Y1, Y2, and Y3 aptamers generated by PEP-Fold. The top row shows the hydrophobicity of amino acid residues, color-coded from white (lowest) to red (highest). The bottom row depicts the electrostatic potential with positive charges represented in blue and negative charges in red. (b) Space-filling and ribbon models of the Dps protein. The Y1, Y2, and Y3 aptamers are genetically attached to the C-terminus of each Dps subunit (indicated by pink dots) *via* an SGGG linker, resulting in the mutants Y1Ct, Y2Ct, and Y3Ct.

We selected CNT-binding peptide aptamers, FKQDAWEAVDIR (Y1), SYTHLLHRSLPG (Y2), SLHNAEMANFRA (Y3), and SHVSWDTKQSGQ (Y4), using the M13 phage display method (Fig. 1).^28^ These amphiphilic peptide sequences contain periodically arranged hydrophobic residues, and such peptides are often reported to adopt α-helical structures, particularly when interacting with hydrophobic interfaces.^29,30^ Y1 and Y2 peptides were genetically attached to the N- and C-termini of Dps in various configurations. Their adsorption was quantitatively analyzed using a quartz crystal microbalance (QCM) with a carbon-coated sensor. The mutant Dps exhibited higher adsorption affinity on the carbon surface than both the wild-type Dps and the Dps modified with carbon nanohorn-binding peptides.^31^ Notably, aptamers attached to the N-terminus of Dps demonstrated superior adsorption efficiency than those attached to the C-terminus, and the insertion of a linker (SGGG) further improved adsorption performance.

In this study, we investigated the binding affinity of Y1, Y2, and Y3 in both their free forms and when attached to Dps. For consistent evaluation under the same conditions in the Dps-attached state, Y1, Y2, and Y3 were genetically attached to the C-terminus of the Dps subunit *via* a linker (SGGG). Y4 was excluded from this analysis due to its absence of structural features resembling α-helices, as predicted in our previous study.^28^ Surprisingly, although free Y3 exhibited low affinity, the Dps modified with Y3 showed remarkably strong adsorption to carbon surfaces. The dissociation constant was a few tenths lower compared to Y1- and Y2-modified Dps. This discovery indicated that the surrounding environment and steric hindrance effects had a significant impact on the binding ability of peptides.

## Experimental material and preparation

### Materials

The potassium hexacyanoferrate(iii) and potassium hexacyanoferrate(ii) trihydrate used in CV measurements were purchased from Wako (Japan). The screen-printed electrodes (SPE) were purchased from Yoshida Co., Ltd (Japan) by special order. The peptide aptamers were synthesized by GL Biochem Shanghai Ltd (Shanghai, China). Ultrapure water (18.2 MΩ cm) was prepared from a Milli-Q system. The PBS was purchased from Gibco (USA).

### Carbon electrode preparation for QCM and CV measurements

For the electrochemical measurement, cyclic voltammetry (CV), carbon screen-printed electrodes (SPE) (Yoshida Co., Ltd, Japan) were used. Each SPE featured a working electrode (WE) with an area of 4 mm^2^, a counter electrode (CE) with an area of 5 mm^2^, and an Ag/AgCl reference electrode (RE) positioned at the center, all of which were integrated onto a single chip (Fig. S2(a)). To ensure fresh electrode surfaces, the SPEs were treated with diamond-lapping for 2 minutes, followed by ultrasonication in ethanol and Milli-Q water for 5 minutes each. Afterward, the electrodes were dried using nitrogen gas and immersed in the PBS with 3 mM [Fe(CN)_6_]^3−/4−^ overnight. A new SPE was used for each protein concentration-dependent measurement to maintain consistency and reliability.

For the QCM measurement, carbon was deposited on a QCM sensor with gold electrodes (shear modulus (SEP), Seiko EG&G, Tokyo, Japan). The sensors were cleaned with Milli-Q water and ethanol at least 3 times, followed by drying with nitrogen gas. After cleaning, carbon deposition was carried out using a vacuum deposition machine (CADE-E). A carbon fiber mesh sheet was used as the carbon source, which could make fine and uniform carbon thin film by vapor deposition. A carbon fiber mesh sheet was placed between the electrodes at the bottom of the vacuum chamber. The QCM sensor was positioned at the top of the vacuum chamber, with a shutter placed in between. Preheating was conducted for 10 seconds to remove surface impurities, after which the shutter was opened, and an electric current of approximately 35 A was applied for Joule heating. Carbon thin films were deposited within 1.7 seconds. Following deposition, the QCM sensors were rinsed with Milli-Q water to remove any undesired carbon film.

### Dps mutant protein preparation

The CNT-binding peptide aptamers (Y1, Y2, and Y3) were genetically attached to the C-terminus of the Dps subunit. The plasmid encoding the modified Dps subunit was transformed into *E. coli*. After cultivation, the *E. coli* cells were harvested, and the resulting pellets were suspended in Tris–HCl buffer. Proteins were extracted through ultrasonic homogenization. The total protein solution was subjected to heat treatment to denature low heat-tolerance proteins, which were subsequently removed by centrifugation. The supernatant containing the target protein was purified using an ion-exchange column (HiTrap™ Q HP 5 mL) with a NaCl ion gradient (0–0.5 M). The purity of the proteins was verified using SDS-PAGE analysis. For further purification, the protein solution underwent gel filtration, sterilization through filtration, and was stored at 4 °C. Before QCM measurements, the protein solutions were prepared in Milli-Q water to ensure consistency and purity.

### 3D structural prediction by PEP-Fold3.5 and AlphaFold3

The aptamer structures were calculated using PEP-Fold3,^32^ a linear peptide prediction tool. This tool is suitable for predicting 3D models of small peptides, ranging from 5 to 50 amino acids. The 3D structure of genetically modified Dps was predicted using the AlphaFold server,^33^ which is based on the AlphaFold3 model code. The amino acid sequence of *Listeria innocua* Clip 11262 Dps was obtained from the Protein Data Bank, and aptamer amino acid sequences, including a linker (SGGG), were added to the C-terminus. These sequences were then input to the AlphaFold server to predict both single-copy and 12-copy structures.^33^ The prediction results showed that the 12-copy structure forms a dodecameric cage-like protein, consistent with the X-ray crystallography results (Fig. S5).

## Results and discussion

### Surface characterization

**Fig. 2:**
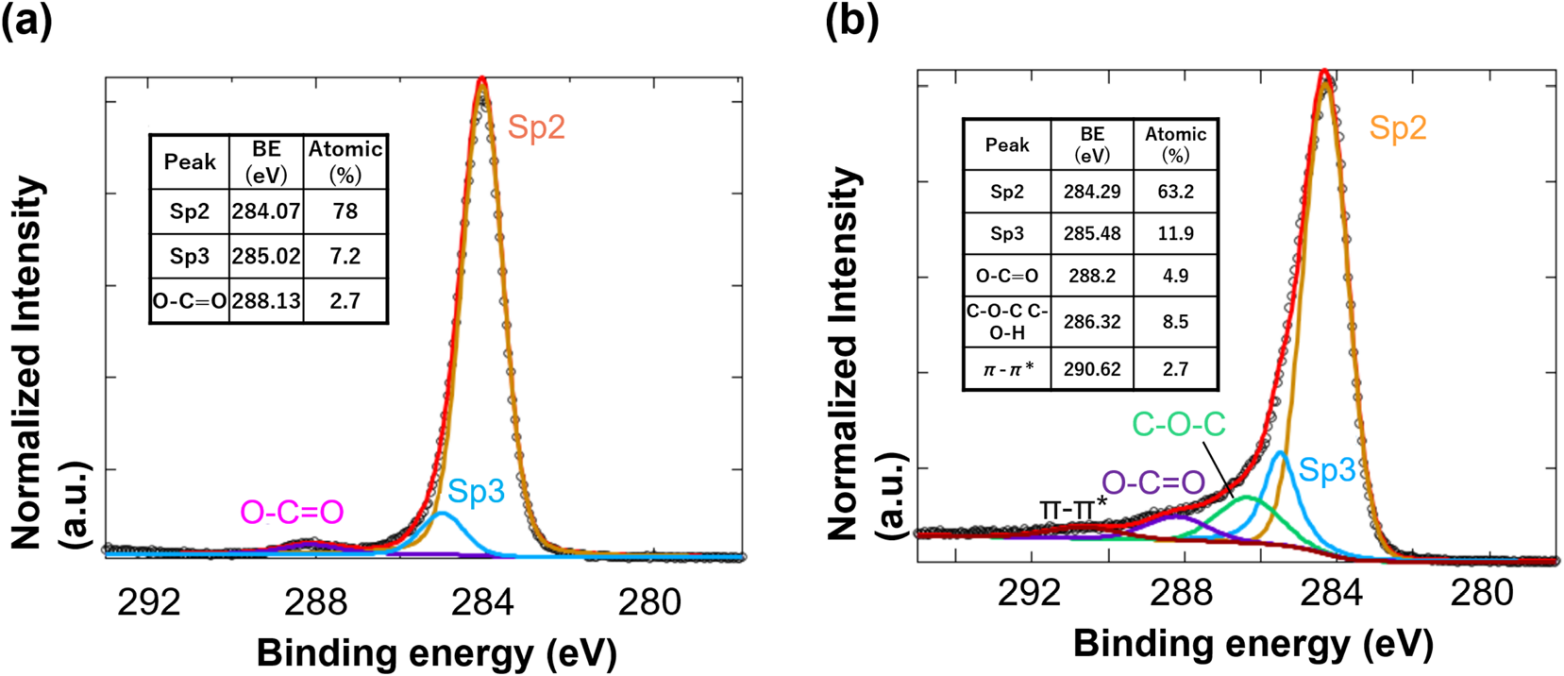
XPS spectra of the C 1s region for (a) screen-printed electrodes (SPE) used in cyclic voltammetry (CV) measurements, (b) carbon-coated QCM sensors. Peak assignments are in the inset table.

The surface properties of the SPE sensors and carbon-coated QCM were analyzed using X-ray photoelectron spectroscopy (XPS, PHI 5000 VersaProbe II, ULVAC-PHI Inc., Kanagawa, Japan). The XPS C 1s spectrum was deconvoluted into the following components: sp^2^ carbon, sp^3^ carbon, carbon with O–C=O bonds, with C–O–C or C–O–H bonds, as shown in Fig. 2. Detailed peak-fitting data were provided in the SI.

The analysis revealed that both the SPE electrodes and carbon-coated sensors were primarily composed of graphite-like sp^2^ bonds, with a minor content of sp^3^ bonds. The SPE electrode exhibited a low degree of oxidation. The QCM sensor contained C–O–C or C–O–H (their binding energies overlap), indicating an oxidized surface (Fig. 2(b)). The higher oxidation of the QCM sensors was likely attributed to the vacuum deposition process, which may have introduced oxygen atoms during carbon deposition.

Further investigation of the carbon bonding states was carried out using Raman spectroscopy (Laser Raman Spectrophotometer, NRS-4100-30, JASCO, Hachioji, Japan). The Raman analysis indicated that the samples had a low crystallinity of carbon material. Detailed results of the Raman analysis were presented in the SI and Fig. S1.

#### Cyclic voltammetry (CV) measurements for the adsorption capacity of free Y1, Y2, and Y3

Cyclic voltammetry (CV) was employed to analyze the adsorption behavior of Y1, Y2, and Y3 peptides on carbon materials owing to its high sensitivity. Freshly cleaned SPE was immersed in PBS (pH 7.4) with 3 mM [Fe(CN)_6_]^3−/4−^ solution, and CV measurements were carried out using an electrochemical analyzer (EmStat Pico Development Kit, PalmSens, Netherlands) operated *via* PSTrace software (Fig. S2(a)).

Initial CV experiments were performed in the absence of peptides or Dps mutants. The scanning potential range was −0.4 V to 0.7 V, with a scan rate of 100 mV s^−1^ and a step size of 2 mV. A total of 11 scan cycles were performed, and data from the final three scans were used to measure the oxidation peak current (*I*_pox_), reduction peak current (*I*_pred_), and peak-to-peak separation (Δ*E*_p_) (see Fig. S2(b)). Three values were averaged to yield the values of *I*_pox_, *I*_pred_, and Δ*E*_p_. Subsequently, Y1 peptide with final concentrations of 1, 3, 10, and 30 µg mL^−1^ was added to the solution. CV measurements were conducted to obtain the values of *I*_pox_, *I*_pred_, and Δ*E*_p_ at each concentration. This process was also performed for Y2 and Y3. The above series of measurements yielded datasets of the concentration-dependent changes in *I*_pox_, *I*_pred_, and Δ*E*_p_ for each peptide aptamer. We conducted this series of experiments six times and averaged the six datasets to obtain the final values for *I*_pox_, *I*_pred_, and Δ*E*_p_.

**Fig. 3:**
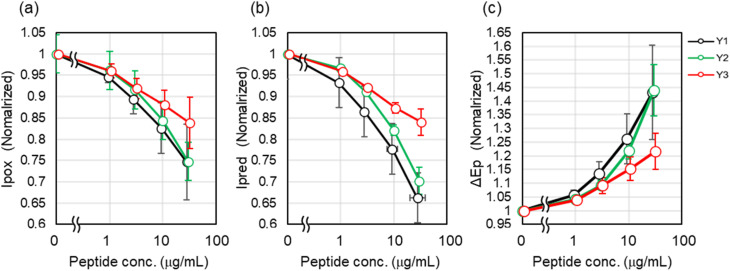
Peptide concentration-dependent changes in (a) *I*_pox_, (b) *I*_pred_, and (c) Δ*E*_p_ obtained from cyclic voltammetry (CV) measurements of Y1, Y2, and Y3 peptides. All data are normalized to initial values, and error bars represent standard deviations.

Fig. 3 presents the results, illustrating the concentration-dependent changes in *I*_pox_, *I*_pred_, and Δ*E*_p_, each normalized to its initial value. In cyclic voltammetry (CV), adsorption of molecules on the electrode surface reduces the effective electroactive area due to surface coverage, leading to a decrease in peak currents (*I*_pox_ and *I*_pred_). Furthermore, adsorption layers hinder interfacial electron-transfer kinetics, resulting in an increase in peak-to-peak separation (Δ*E*_p_). Such behavior is well established for surface-blocking processes in electrochemical systems.^34^ Accordingly, as the concentration of the peptide aptamers increased, *I*_pox_ and *I*_pred_ decreased, while Δ*E*_p_ widened. All concentration-dependent changes in *I*_pox_, *I*_pred_, and Δ*E*_p_ showed the same trend. Y1 exhibited the most significant change, followed closely by Y2, while Y3 exhibited smaller changes. These results indicated that Y1 and Y2 had similar adsorption affinities, whereas Y3 exhibited a lower adsorption affinity.

Our previous research predicted that peptides Y1, Y2, and Y3 would adopt α-helical structures. Furthermore, previous reports have shown that amphiphilic peptide sequences with periodic hydrophobic residues tend to form α-helical structures on hydrophobic surfaces.^29,30^ Therefore, it is reasonable to consider that Y1 and Y2 form amphipathic α-helical structures, facilitating adsorption onto carbon surfaces through hydrophobic interactions (see Fig. 1).^28^ Specifically, Y1 and Y2 were anticipated to display hydrophobic amino acids predominantly on one side of the helix, facilitating adsorption onto carbon surfaces through hydrophobic interactions. In contrast, although Y3 was also predicted to form an α-helix, the distribution of its hydrophobic amino acids was less pronounced, suggesting a weaker hydrophobic interaction with carbon surfaces and, consequently, lower adsorption ability in its free state. The CV results were consistent with these structural and interaction-based predictions.

#### Cyclic voltammetry (CV) measurements for the adsorption capacity of Y1-, Y2-, and Y3-modified Dps

**Fig. 4:**
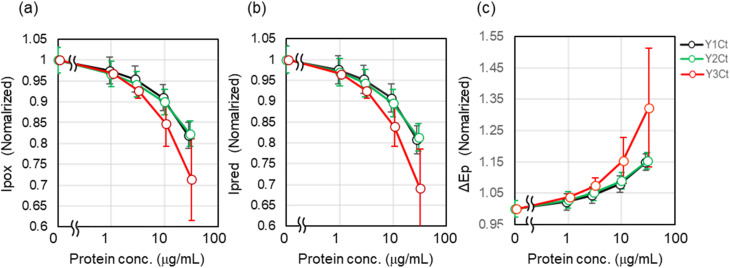
Concentration-dependent changes in *I*_pox_, *I*_pred_, and Δ*E*_p_ obtained from CV measurements of Y1Ct, Y2Ct, Y3Ct. (a) *I*_pox_, (b) *I*_pred_, and (c) Δ*E*_p_. All data are normalized to initial values, and error bars represent standard deviations.

We genetically attached Y1, Y2, and Y3 peptides to the C-termini of Dps with a linker (SGGG), which were designated Y1Ct, Y2Ct, and Y3Ct, respectively. The binding abilities of these Dps mutant proteins were evaluated using CV. Surprisingly, although the free Y3 peptide exhibited low adsorption affinity, Y3Ct showed changes in *I*_pox_, *I*_pred_, and Δ*E*_p_ that were approximately twice as large as those of Y1Ct and Y2Ct, demonstrating its superior adsorption capability to Y1Ct and Y2Ct. The relative adsorption affinities of Y1/Y2 and Y3 were reversed upon immobilization. These findings demonstrated that the adsorption ability of Y3 was significantly enhanced upon immobilization on the Dps surface (Fig. 4).

The nearly identical adsorption behavior observed for Y1Ct and Y2Ct suggested that they shared the same adsorption mechanism. As described above, Y1 and Y2 were experimentally shown to possess comparable adsorption abilities; thus, the adsorption of Y1Ct and Y2Ct was inferred to arise from Y1 and Y2 peptides presented on the Dps surface, which adopted α-helical conformations and adsorbed onto the carbon surface *via* hydrophobic interactions. In contrast, the strong adsorption ability of Y3Ct could not be explained solely by hydrophobic interactions, since free Y3 exhibited only weak hydrophobic interactions. These findings suggested the involvement of an additional or distinct adsorption mechanism.

#### QCM batch-mode measurement of the adsorbed mass for Y1-, Y2-, and Y3-modified Dps proteins

To obtain quantitative data on adsorption behavior, quartz crystal microbalance (QCM) measurements were employed. Although QCM is unsuitable for small peptides due to their low mass, it is applicable to mutant Dps (∼10 nm in size), allowing a more accurate assessment of their adsorption capacity. We employed two QCM measurement methods: batch mode and open-flow mode (Fig. S3).

In the batch-mode, changes in resonance frequency (Δ*F*) were intermittently monitored. The temperature of the chamber was maintained at 25 ± 0.1 °C to ensure stable operation. Data were collected at 27 MHz (overtone number 3) using PS-P700/W32 WinQCMA software (Seiko EG&G, Tokyo, Japan). We filled the chamber with Milli-Q water and established the baseline frequency, then introduced the protein solution (0.1 mg mL^−1^). The Dps mutants adsorbed to the sensor, and the resonance frequency decreased. The solution was then removed, and the sensor was washed with Milli-Q water to remove weakly adsorbed proteins. The chamber was then filled with Milli-Q water, and measurement was resumed. The decrease in resonance frequency from the baseline obtained at this time was attributed to the strongly adsorbed mass of the Dps mutant. The amount of adsorbed protein could be calculated from this Δ*F* using the Sauerbrey equation. (For details of the experiment, see SI).

**Fig. 5:**
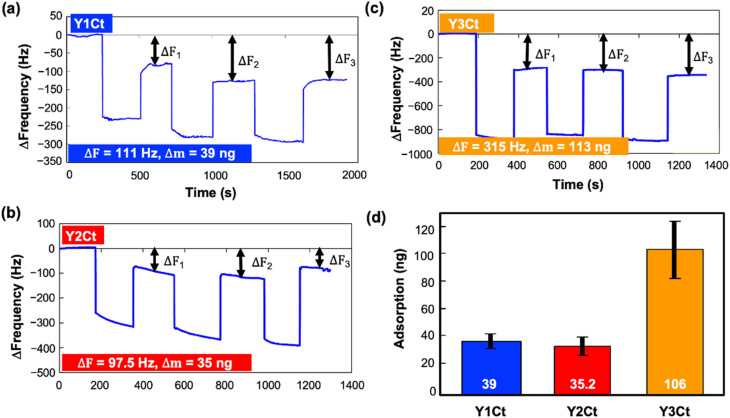
Typical resonance frequency shifts (Δ*F*) over time during three cycles of protein injection and removal at a concentration of 0.1 mg mL^−1^ for (a) Y1Ct, (b) Y2Ct, and (c) Y3Ct. (d) The averaged adsorbed mass with standard deviation for Y1Ct, Y2Ct, and Y3Ct, determined by 30 QCM batch-mode experiments.

The above process was repeated three times in one measurement. Each sensor was used only once for each measurement. Typical changes in resonance frequency (Δ*F*) for Y1Ct, Y2Ct, and Y3Ct were shown in Fig. 5(a, b and c). This three-times measurement was repeated 10 times for each Dps mutant, for a total of 30 adsorption measurements. All calculated adsorbed mass (Δ*m*) was listed in the SI in Table S1. The average adsorption mass of Y1Ct, Y2Ct, and Y3Ct measured was 39 ng (183 ng cm^−2^), 35.2 ng (179 ng cm^−2^), and 106 ng (541 ng cm^−2^), respectively (Fig. 5(d)). Y3Ct exhibited an adsorption amount approximately three times more than that of Y1Ct and Y2Ct. The trend in the difference in adsorbed mass was consistent with the difference observed in CV measurements, and the adsorbed mass was quantitatively measured.

#### Open-flow measurement of dissociation constants of Y1-, Y2-, and Y3-modified Dps proteins

We further evaluated the dynamic reactions and adsorption rates of these Dps mutants by open-flow mode equipment (QCM934-500, Seiko EG&G, Tokyo, Japan) to quantitatively assess their adsorption capacity. Dps molecules were well dispersed in aqueous solution without observable aggregation. Adsorption was considered to occur predominantly at the level of individual molecules.

The sensor was first stabilized in Milli-Q water, after which protein solutions were introduced at a flow rate of 50 µL min^−1^. The change in resonance frequency (Δ*F*) relative to the stabilized initial frequency was then recorded continuously. As a control, measurements were performed using native (wild-type) Dps. Introduction of native Dps at concentrations of 0.1 and 0.2 mg mL^−1^ resulted in negligible changes in resonance frequency (see SI, Fig. S4). Measurements were then conducted by introducing Y1Ct and Y2Ct at final concentrations of 0.05, 0.1, 0.25, 0.5, 0.75, and 1 mg mL^−1^. For Y3Ct, due to its significantly higher binding affinity, measurements were conducted at lower concentrations of 0.005, 0.01, 0.025, 0.05, 0.075, and 0.1 mg mL^−1^ to avoid early saturation and to accurately capture the adsorption behavior.

**Fig. 6:**
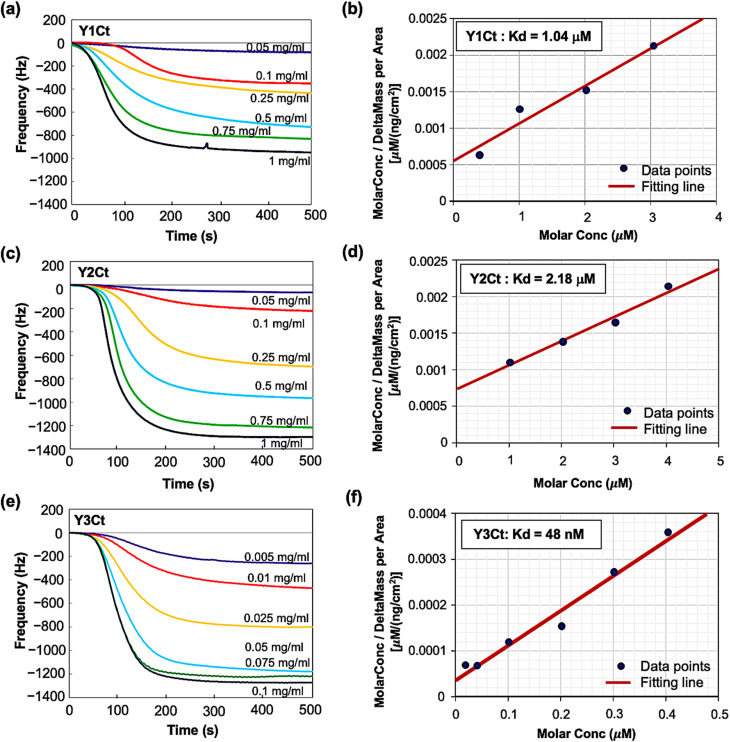
QCM open-flow measurements and *K*_d_ determination: (a and b) QCM results and *K*_d_ determination for Y1Ct, (c and d) for Y2Ct, (e and f) for Y3Ct. Linear fitting using the least-squares method is shown, with black dots representing each data point at varying concentrations. As a control experiment, open-flow QCM measurements using native (wild-type) Dps were performed under identical conditions, showing negligible frequency change (see Fig. S4 in SI).

The Δ*F* decreased following an exponential saturation model: Δ*F* = *a*·(1 − exp[−*t*/*b*]) + *c* (Fig. 6(a), (c) and (e)). The response curves exhibited gradual, minor changes at low protein concentrations, transitioning to sharp, significant declines at higher concentrations. Notably, for Y3Ct, a pronounced decrease in Δ*F* was observed starting at 0.005 mg mL^−1^. In contrast, even at a tenfold higher concentration (0.05 mg mL^−1^), Y1Ct and Y2Ct exhibited only minimal decreases in Δ*F*. The dissociation constant (*K*_d_) was determined through linear fitting using eqn (1), as detailed in the SI.1
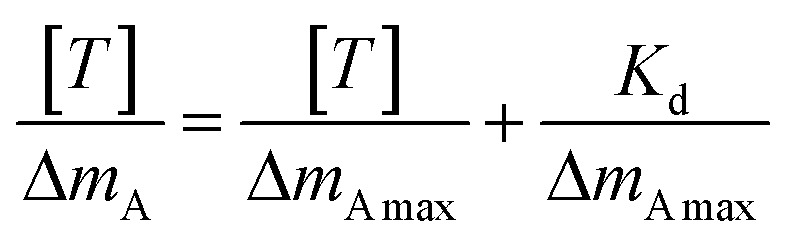
where *M*_w_ – the molecular weight of Dps, [*T*] – initial concentration of Dps, *V* – the volume of the reaction system, *S* – the electrode area, Δ*m*_A_ – amount of bind protein per unit area [ng cm^−2^], Δ*m*_Amax_ – maximum binding capacity per unit area [ng cm^−2^], *K*_d_ – dissociation constant [*M*].

Eqn (1) can be analyzed by plotting 
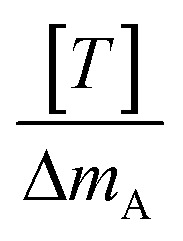
 on the vertical axis and [*T*] on the horizontal axis. By fitting the data with a linear function, the *y*-intercept yielded the value of 
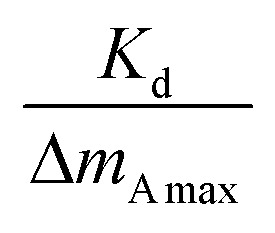
, which was subsequently used to calculate *K*_d._ The amount of bound protein (Δ*m*_A_) and maximum binding capacity (Δ*m*_Amax_) were calculated using QCM software (PS–P700/W32 WinQCMA, Seiko EG&G, Tokyo, Japan). Fig. 6(b), (d) and (f) illustrate the linear fitting using eqn (1). As shown in the figures, the fitting quality was excellent. The dissociation constants (*K*_d_) calculated were as follows: for Y1Ct, *K*_d_ = 1.04 µM (*R*^2^ = 0.95); for Y2Ct, *K*_d_ = 2.18 µM (*R*^2^ = 0.98), and for Y3Ct, *K*_d_ = 48 nM (*R*^2^ = 0.97). The fitting yielded high determination coefficients (*R*^2^).

As a result of the numerical evaluation, the dissociation constants of each Dps mutant were determined, allowing for a precise comparison of the adsorption capabilities among Y1Ct, Y2Ct, and Y3Ct. The results revealed that the dissociation constant, *K*_d_, for Y3Ct was significantly lower than those for Y1Ct and Y2Ct. Specifically, the *K*_d_ of Y3Ct was approximately 20 to 45 times smaller than that of Y1Ct and Y2Ct, indicating significantly higher binding affinity. Notably, while the free Y3 peptide aptamer exhibited a low affinity for the carbon surface, its immobilization on the Dps surface resulted in a substantial increase in adsorption affinity. Additionally, the determined *K*_d_ values highlighted clear differences in adsorption affinities between Y1Ct and Y2Ct, distinctions that were previously ambiguous in batch-mode measurements and cyclic voltammetry (CV) measurements.

#### Discussion on immobilization-driven enhancement of CNT-binding peptide affinity

Previous studies have reported that even minor modifications to an aptamer can significantly alter its three-dimensional structure and functions.^35,36^ In this study, we showed that the adsorption capacity of Y3 was markedly enhanced when immobilized on the Dps surface. In contrast, Y1 and Y2 did not exhibit substantial changes in adsorption behavior upon immobilization, and it was inferred that their α-helical-like structures adsorbed to the carbon surface through hydrophobic amino acids localized on one face of the helix.^28^ To investigate these differences, we first predicted the conformations of the immobilized aptamers using AlphaFold3 based on their amino acid sequences.

**Fig. 7:**
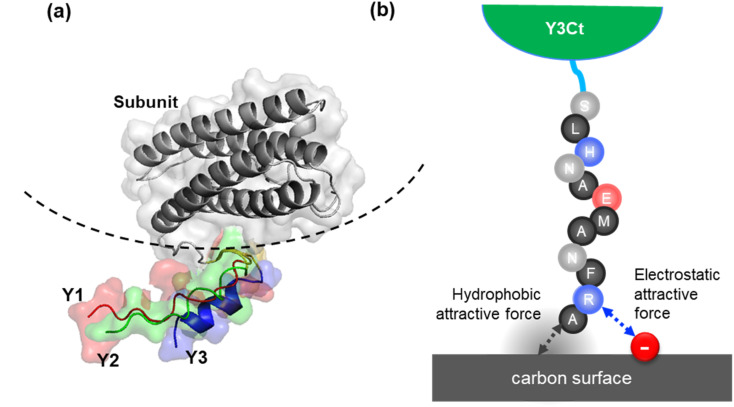
(a) Predicted model of the Dps subunit/linker/aptamer structure generated by AlphaFold3. (b) A schematic illustration of a plausible mechanism for the enhanced adsorption of Y3Ct on carbon surfaces.

The AlphaFold3 prediction for the Dps monomer (four-helix bundle region) closely matched the X-ray crystallographic structure (6QHV PDB), with a Root Mean Square Deviation (RMSD) of 0.158 Å, demonstrating high accuracy (Fig. S5(a)). The attached aptamers adopted generally similar conformations. Y1 and Y2 formed disordered α-helices aligned along the Dps surface, whereas Y3 formed an α-helix slightly skewed outward, with its C-terminus oriented away from the Dps surface (Fig. 7(a) and S5).

Since Y1Ct and Y2Ct exhibited nearly identical adsorption behaviors, their binding was presumed to be governed by hydrophobic interactions, as in the free state. Because the aptamers were immobilized on Dps through their N-termini, we inferred that the C-terminal region of Y3 played a distinct role in the enhanced adsorption.

Several factors could contribute to adsorption, including electrostatic interactions, spatial constraints, and cooperative binding of multiple peptide aptamers. Among these, electrostatic interactions appear the most plausible. For example, the selective adsorption of a Ti-binding hexapeptide has been elucidated to occur through electrostatic interactions between the N-terminal arginine and the sixth-position aspartic acid with the surface charges of Ti.^14^

Based on the structural differences, we propose a plausible mechanism for the enhanced adsorption of Y3Ct. The outward orientation of the Y3 C-terminus may increase the accessibility of positively charged residues (*e.g.*, arginine near the C-terminus), enabling electrostatic interactions with negatively charged regions on the carbon surface (Fig. 7(b)). In addition, multivalent presentation of Y3 peptides on the Dps cage may promote cooperative binding, further stabilizing adsorption.

It should be noted that the predicted structures carry uncertainty. The pLDDT analysis (Fig. S5 and Table S2) indicates high confidence in the Dps core (pLDDT > 90), but lower confidence in the linker and aptamer regions (∼25–55), suggesting structural flexibility. Therefore, the proposed mechanism should be regarded as a plausible interpretation rather than a definitive conclusion. Further experimental and analytical studies—such as ionic strength dependence or site-directed mutagenesis—will be required to validate and refine this mechanism.

## Conclusions

We investigated the adsorption behavior of aptamers Y1, Y2, and Y3 onto carbon surfaces in both their free state and when displayed on the cage-shaped protein Dps, using electrochemical measurements and QCM. In the free state, Y1 and Y2 exhibited comparable adsorption capacities, whereas Y3 showed lower adsorption. In contrast, Dps mutant proteins displaying Y3 on their outer surface demonstrated the highest adsorption ability, with a dissociation constant (*K*_d_) as low as 48 nM, approximately several tens of times lower than that of the Y1- and Y2-modified Dps mutants. These results clearly indicate that the binding affinity of the Y3 aptamer is significantly enhanced upon presentation on the Dps protein surface, highlighting the significant impact of immobilization on aptamer performance. Structural analysis suggests that the orientation of the C-terminal region of Y3 may contribute to enhanced adsorption, potentially through increased accessibility and multivalent interactions involving multiple peptide termini. However, further experimental studies will be required to fully elucidate the underlying mechanism. These findings provide new insights into immobilization-driven modulation of peptide-nanomaterial interactions and offer a basis for the rational design of bio-nanomaterial interfaces for advanced nanodevices.

## Conflicts of interest

The authors declare no conflict of interest.

## Supplementary Material

RA-016-D5RA09758D-s001

## Data Availability

The data supporting the findings of this study are available within the article and its supplementary information (SI). Supplementary information: Raman experimental data, CV measurement data, QCM experimental procedures and analyses, control QCM measurements using native wild-type Dps, batch- and open-flow-mode data analyses including Δ*m* and *K*_d_ calculations, AlphaFold3-predicted structures of the Dps subunit/linker (SGGG)/aptamer constructs, and XPS analysis. See DOI: https://doi.org/10.1039/d5ra09758d.
