# Supplementary material for: Immobilization-driven enhancement of CNT-binding peptide affinity: substantial shift from the free-state
Source: RSC Adv. 2026 May 8;16(26):24174–82. doi: 10.1039/d5ra09758d (PMC13154780; doi:10.1039/d5ra09758d)
Supplement: RA-016-D5RA09758D-s001 [file RA-016-D5RA09758D-s001.pdf]

Supplementary Information (SI) for RSC Advances.
This journal is © The Royal Society of Chemistry 2026

Supporting Information

Contents:

1. Raman experiment
2. CV measurement
3. QCM experiments procedure
4. Batch-mode data analysis: Δm calculation
5. Control experiment: Open-flow QCM measurement of native (wild-type) Dps
6. Open-flow-mode data analysis: Dissociation constant (K_d) calculation
7. The monomeric and 12-mer assembly structures of the Dps subunit/linker (SGGG)/aptamer (Y1, Y2, and Y3) predicted by the AlphaFold3
8. XPS analysis

1. *Raman experiment*

Raman spectroscopy measurements were conducted using a Laser Raman Spectrophotometer (NRS-4100-30, JASCO) with a 532 nm excitation laser and a beam diameter of 1 μm. Spectra were acquired from multiple locations on the sample to confirm reproducibility, and the results showed consistent spectral profiles across all measured points. A typical Raman spectrum is presented in Figure S1(a).

The observed spectrum showed a broad peak (highlighted in red), with the G and D bands characteristic of carbon materials not distinctly resolved. This broadening was attributed to the low crystallinity of the carbon layer, indicating a distorted crystal structure with numerous defects. To further evaluate the carbon structure, peak deconvolution of the broad spectrum was performed, isolating the G and D bands (shown in blue).□The G band was observed at approximately 1524 cm^{-1}, while the D band appeared at 1362 cm^{-1}. The intensity of the G band was slightly higher than that of the D band, indicating that the surface of the carbon layer was marginally dominated by sp^2-bonded carbon, suggesting that the carbon structure with sp^2 bonds was more abundant than sp3 at the top surface of the carbon film.

Figure S1(b) shows the Raman spectrum of SPE, where the G and D bands overlapped but appeared separated. This indicated that the SPE was more crystalline than the QCM carbon film.

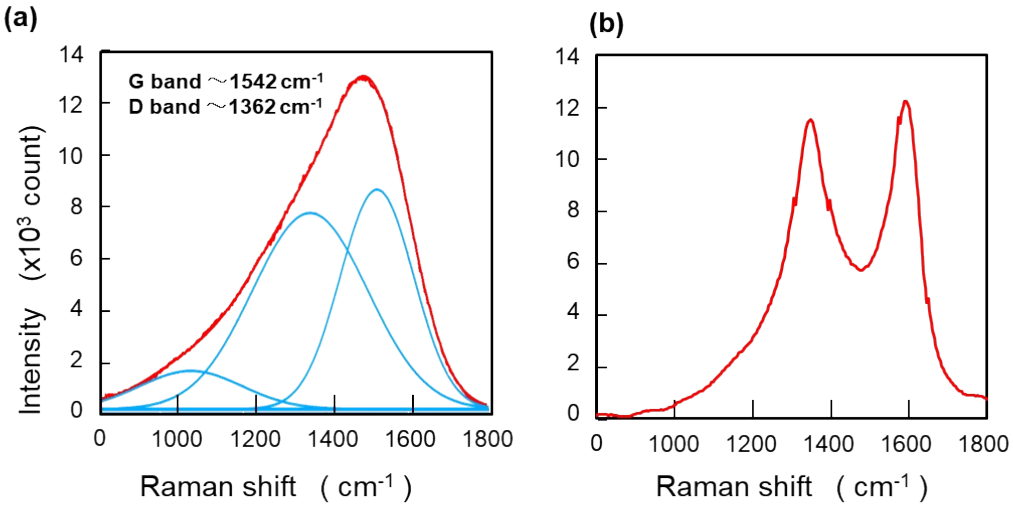

Figure S1. Raman spectra of (a) carbon coated QCM sensors, (b) SPE.

2. *CV measurement*

Cyclic voltammetry (CV) measurements were conducted using a screen-printed electrode (SPE; Yoshida Co., Ltd.) and an electrochemical analyzer (EmStat Pico Development Kit, PalmSens, Netherlands) operated via PSTrace software. The configuration of the carbon SPE is depicted in Figure S2(a). The SPE featured a working electrode (WE) area of 4 mm^2, a counter electrode (CE) area of 5 mm^2, and an Ag/AgCl reference electrode (RE) positioned at the center.

Prior to measurements, the SPE surfaces were prepared by diamond-lapping for 2 minutes, followed by ultrasonication in ethanol and Milli-Q water for 5 minutes each. The electrodes were then dried under nitrogen gas. The electrochemical measurement setup is shown in Figure S2(a). The SPE was immersed in a PCR tube containing 100 µL of PBS with 3 mM $[Fe(CN)_6]^{3-/4-}$ solution.

Initial CV experiments were performed in the absence of peptides or mutant Dps. The scanning potential range was -0.4 V to 0.7 V, with a scan rate of 100 mV/s and a step size of 2 mV. A total of 11 scan cycles were performed, and data from the final three scans were used to calculate the oxidation peak current (I_{pox}), reduction peak current (I_{pred}), and

peak-to-peak separation (ΔE_p) (See Figure S2(b)). These values were averaged to yield final measurements for I_{pox}, I_{pred}, and ΔE_p.

Subsequently, a predetermined amount of peptide or mutant Dps was added to the solution, and CV measurements were performed to determine I_{pox}, I_{pred}, and ΔE_p. This process was repeated for each peptide and protein to obtain data on the concentration-dependent changes in I_{pox}, I_{pred}, and ΔE_p. As an example, a series of the 11 cycles of CV measurements for Y3Ct when the protein concentration was changed at 0, 1, 3, 10, and 30 µg/mL are superimposed and shown in Figure S2 (c).

For each peptide aptamer, six independent concentration-dependent experiments were conducted, and four replicate measurements were performed for each mutant Dps protein. The average of these measurements was used as the final dataset. The observed decrease in peak currents and the increase in peak-to-peak separation were attributed to the adsorption of peptide aptamers or mutant Dps onto the electrode surface. A larger magnitude of these changes corresponded to a higher degree of adsorption. Consequently, these measurements enabled the determination of the relative affinity of each peptide aptamer or mutant Dps for the electrode surface.

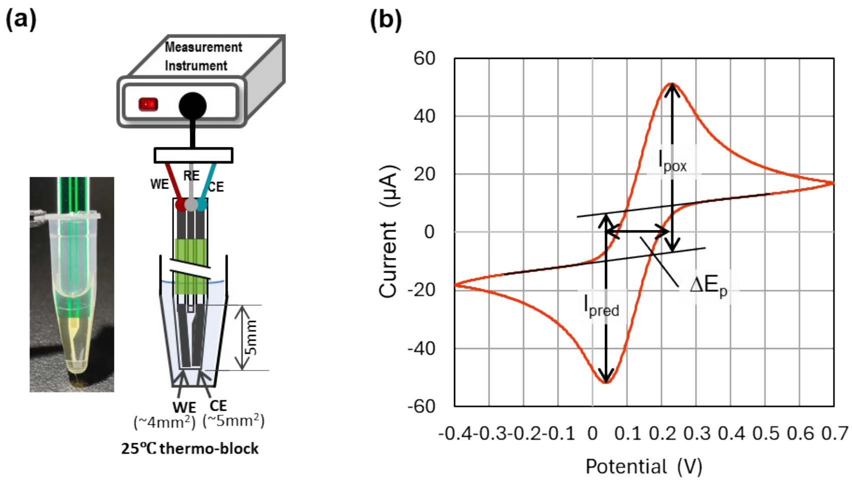

(c)

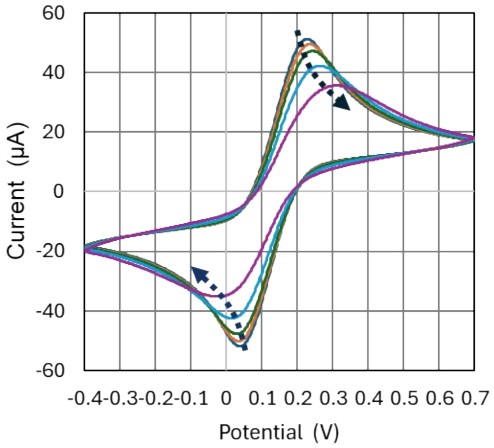

Figure S2. (a) Cyclic voltammetry (CV) measurement setup utilizing the screen-printed electrode (SPE), and (b) CV graph obtained with the SPE in a PBS solution containing 3 mM $[Fe(CN)_6]^{3-/4-}$, showing the measurements of oxidation peak current (I_{pox}), reduction peak current (I_{pred}), and the peak-to-peak separation (ΔE_p). (c) A series of CV measurements of Y3Ct. Protein concentrations were 0, 1, 3, 10, and 30 μg/mL along the dashed arrow.

3. *QCM experiments procedure*

To investigate the adsorption and binding behavior of cage-shaped proteins on a carbon surface, the QCM measurement was carried out in situ and in real time using a QCM934 with a QA-CL4 well-type cell, made by Seiko EG&G. Quartz is one member of a family of crystals that experience the piezoelectric effect. Applying alternating current to the AT-cut quartz crystal will induce oscillations, and a standing shear wave is generated. (Figure S3) Based on this physical resonance phenomenon, QCM is a mass loading sensor and a powerful technique which detects the mass of molecules adsorbed on a sensor. As mass is adsorbed, the weight increases; consequently, the resonance frequency decreases from the initial value. Molecular adsorption introduces not only a mass change but also dissipation (ΔD) of mechanical energy due to the internal friction of the adsorbed layer. In this work, however, dissipation was not evaluated due to instrumental limitations, and thus, the viscoelastic properties of the adsorbed layer were not analyzed.

This study quantitatively characterizes the adsorption behavior of the target biomolecules, including their adsorbed amounts and kinetics, using two straightforward

QCM measurement modes: batch mode and open-flow mode. Commercially available gold-coated QCM sensors (QA-A9M-AU(M)(SEP), Seiko EG&G, Tokyo, Japan) were used for the experiments. Each sensor was used only once for each measurement. The sensor and chamber temperature were controlled at 25 ± 0.1 °C to ensure stable operation. Data were collected at 27 MHz (overtone number 3) and analyzed using PS-P700/W32 WinQCMA software (Seiko EG&G).

In the QCM batch-mode measurements, changes in the resonance frequency (ΔF) (Δd) were monitored intermittently. The experiment commenced by filling the measurement chamber with Milli-Q water. Once the resonance frequency stabilized, this value was recorded as the protein-free baseline. A mutant Dps protein solution at a concentration of 0.1 mg/mL was then introduced into the chamber, allowing the protein to adsorb onto the carbon surface. This adsorption process reduced the resonance frequency relative to the baseline. After the frequency stabilized, the measurement was paused, and the protein solution was removed. Milli-Q water was subsequently introduced into the chamber to wash away weakly adsorbed Dps proteins from the sensor surface, and the measurement was resumed.

This cycle of protein adsorption followed by washing was repeated three times. The change in resonance frequency relative to the baseline after each washing step (ΔF) was recorded and used to calculate the mass of strongly adsorbed mutant Dps. A schematic representation of a typical frequency change is provided in Figure S3(b). Each sensor was utilized for a single set of three adsorption cycles. This three-cycle measurement protocol was repeated 10 times for each mutant Dps, resulting in a total of 30 adsorption measurements per mutant.

In the open-flow-mode experiments, the QCM system was also equilibrated with Milli-Q water prior to measurements. The sensor was rinsed with Milli-Q water at a constant flow rate of 50 μL/min using a peristaltic pump. This process stabilized the resonant frequency to a level not exceeding 0.5 Hz for at least one hour, ensuring the stability and reliability of the experiment. After the stabilization, the protein solution was introduced with a final concentration of 0.05 mg/mL to 1 mg/mL. The frequency change was recorded continuously until it stabilized. For the open-flow measurement, no rinsing was carried out. (Figure S3(c)) Further, Kd of molecules on the surface was kinetically evaluated from the open-flow experiment results.

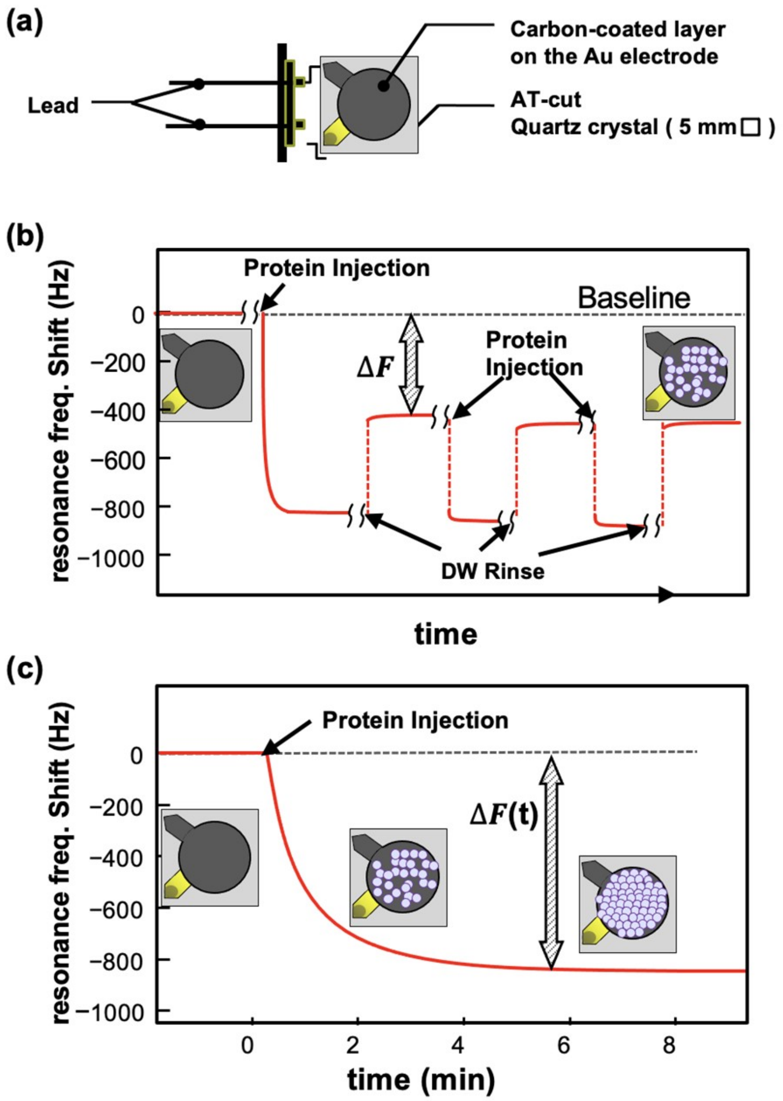

Figure S3. (a) Crystal sensor of QCM934. (b) Typical profile of frequency changes in the batch-mode measurement. The changes in frequency, ΔF, are defined as the difference between the baseline frequency and the frequency after rinsing. (c) Typical profile of frequency changes in the open-flow measurement. In this measurement, the binding affinity of molecules on the surface can be kinetically evaluated using several measurements with different concentrations.

4. Batch-mode data analysis: Δm calculation

The mass (Δm) of the strongly adsorbed molecules per unit area was calculated using the Sauerbrey equation (Eq. 1) from the 30 ΔF values obtained from each mutant Dps measurement.

Sauerbrey equation

$$\Delta F = -\frac{2}{\sqrt{\mu\rho}} \times \frac{F^2}{n} \times \frac{\Delta m}{S} \quad (1)$$

where the terms are defined as follows:

ΔF: frequency change (Hz);

F: measurement frequency (F = 27 MHz in this work);

Δm: mass change (g);

ρ: density of quartz (ρ = 2.648 g/cm^3);

S: piezoelectrically active crystal area (area cm^2);

μ: shear modulus of quartz for AT-cut crystal ($\mu = 2.947 \times 10^{11}$ g/(cm·s^2));

n: overtone number ($n = 3$ in this work).

Using an AT-cut quartz crystal with a fundamental frequency of 9 MHz and an electrode diameter of 5 mm, measurements were conducted at 27 MHz (overtone number n = 3). The sensitivity in this study was approximately -0.36 ng/Hz (approximately -1.8 ng/Hz/cm^2). All ΔF values and their corresponding calculated masses (Δm) are summarized in Table S1, with the average value presented at the bottom.

Significant differences in adsorption behavior were observed among Y1Ct, Y2Ct, and Y3Ct. To ensure the reliability of the results and minimize experimental variability, the batch-mode experiment was repeated ten times, and the average of 30 ΔF measurements was used for adsorption quantification. The average ΔF values for Y1Ct, Y2Ct, and Y3Ct were 109.3 Hz, 98.5 Hz, and 297 Hz, respectively. These frequency changes were converted into mass changes (Δm) using the Sauerbrey equation (Equation 1), 39 ng (183 ng/cm^2) for Y1Ct, 35.2 ng (179 ng/cm^2) for Y2Ct, and 106 ng (541 ng/cm^2) for Y3Ct,

respectively. These results, summarized in Figure 5 of the main text, clearly demonstrate the superior adsorption capacity of Y3Ct.

Table S1. All ΔF and Δm obtained by the QCM batch-mode measurements.

	Y1Ct		Y2Ct		Y3Ct	
	ΔF [Hz]	Δm [ng]	ΔF [Hz]	Δm [ng]	ΔF [Hz]	Δm [ng]
1	80.84	28.9	103.64	37.0	289.53	103.4
2	122.5	43.7	130.83	46.7	263.58	94.1
3	122.33	43.7	188.9	67.4	270.65	96.6
4	77.27	27.6	91.79	32.8	324.98	116.0
5	128.37	45.8	100.84	36.0	315.62	112.7
6	178.63	63.8	91.72	32.7	359.09	128.2
7	91.71	32.7	103.66	37.0	293.99	105.0
8	97.37	34.8	127.83	45.6	304.29	108.6
9	91.8	32.8	119.56	42.7	348.45	124.4
10	87.68	31.3	93.26	33.3	379.66	135.5
11	77.94	27.8	117	41.8	375.42	134.0
12	99.56	35.5	82.52	29.5	393.19	140.4
13	90.06	32.2	84.41	30.1	310.05	110.7
14	120.01	42.8	93.5	33.4	310.55	110.9
15	99.26	35.4	80.82	28.9	248.12	88.6
16	95.78	34.2	97.67	34.9	166.95	59.6
17	139.15	49.7	141.96	50.7	243.41	86.9
18	125.31	44.7	102.97	36.8	174.22	62.2
19	86.2	30.8	73.12	26.1	175.83	62.8
20	94.76	33.8	87.79	31.3	201.77	72.0
21	105.59	37.7	101.71	36.3	201.65	72.0
22	108.52	38.7	69.06	24.7	323.53	115.5
23	133.73	47.7	95.54	34.1	332.8	118.8
24	99.81	35.6	85.49	30.5	327.71	117.0
25	130.67	46.6	79.17	28.3	304.33	108.6
26	130.64	46.6	79.85	28.5	347.61	124.1
27	113.89	40.7	83.42	29.8	339.91	121.3
28	108.31	38.7	82.93	29.6	363.8	129.9
29	126.78	45.3	81.2	29.0	302.75	108.1
30	114.12	40.7	82.8	29.6	318.34	113.6
Average	109.3	39.0	98.5	35.2	297.1	106.1

5. Control experiment: Open-flow QCM measurement of native (wild-type) Dps

Prior to the open-flow QCM measurements of Dps mutants, a control experiment was conducted using native (wild-type) Dps under identical experimental conditions (QCM934-500, Seiko EG&G, Tokyo, Japan). The sensor was first stabilized in Milli-Q water, after which protein solutions were introduced at a flow rate of 50 μL/min. As a control, wild-type Dps was introduced at final concentrations of 0.1 and 0.2 mg/mL in two independent measurements. The results are shown in Figure S4. A sufficiently long baseline prior to protein introduction is shown to confirm the stability of the sensor. In both cases, no significant change in resonance frequency was observed upon addition of wild-type Dps, and only minimal fluctuations were detected. These results indicate that wild-type Dps exhibits negligible adsorption onto the carbon surface.

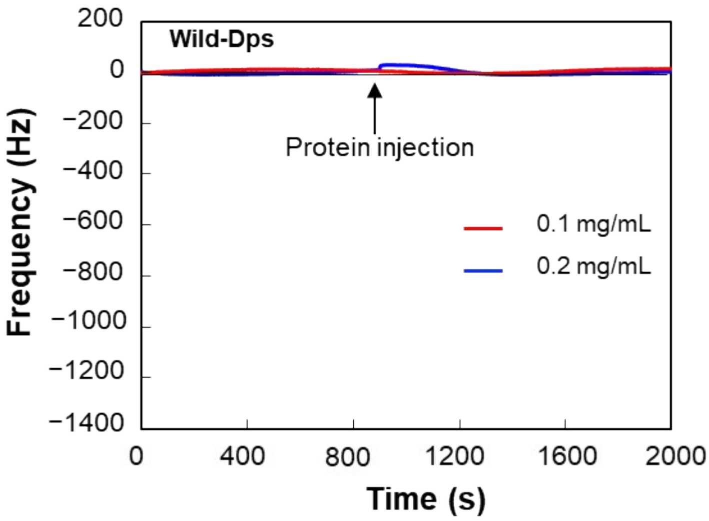

Figure S4. Open-flow QCM response of native (wild-type) Dps under identical experimental conditions as those used for Dps mutants.

6. Open-flow-mode data analysis: Dissociation constant (K_d) calculation

To quantitatively evaluate the adsorption of mutant Dps on carbon substrates, the dissociation constant (Kd) was calculated. The Kd is defined as follows using the concentration of unreacted A, B and the complex AB.

$$K_d = \frac{[A][B]}{[AB]} \qquad (2)$$

In the case of Dps adsorption, A is the electrode surface, B is the Dps, and AB is Dps bound on the carbon surface. Kd was calculated by the following equations.

$$[AB] = \frac{\Delta m_A \, S}{Mw \, V} \qquad (3)$$

$$[B] = [T] - \frac{\Delta m_A \, S}{Mw \, V} \qquad (4)$$

$$[A] = \frac{(\Delta m_{Amax} - \Delta m_A)\, S}{Mw \, V} \qquad (5)$$

$$Kd = \frac{(\Delta m_{Amax} - \Delta m_A)\,([T] - \frac{\Delta m_A \, S}{Mw \, V})}{\Delta m_A} \qquad (6)$$

where

Mw - The molecular weight of Dps,

[T] - Initial concentration of Dps,

V - The volume of the reaction system,

S - The electrode area,

Δm_A – Amount of binding per unit area [ng/cm^2],

Δm_{Amax} - Maximum binding amount (or saturated) per unit area [ng/cm^2],

K_d - Dissociation constant [M]

In our measurements, $\frac{(\Delta m_{Amax} - \Delta m_A)\, S}{Mw \, V}$ is negligibly small, so the dissociation constant K_d can be expressed as follows:

$$Kd = \frac{(\Delta m_{Amax} - \Delta m_A)\,[T]}{\Delta m_A} \qquad (7)$$

Further, this relationship can be reformulated as follows.

$$\frac{[T]}{\Delta m_A} = \frac{[T]}{\Delta m_{Amax}} + \frac{K_d}{\Delta m_{Amax}} \qquad (8)$$

Using equation (8) and the fitting method, we derived the Kd. The change in the resonance frequency of the flow mode theoretically becomes the response expressed in Equation (9), where a, b, and c are constants, and t is the time.

$$\Delta f = a \cdot (1 - \exp[-t/b]) + c \qquad (9)$$

The constants in equation (9) were determined by fitting the data points obtained at each protein concentration to equation (9). The saturated Δf for each protein concentration was determined. The saturated Δf can be converted to the saturated Δm_A using the Sauerbrey equation. After Δm_A values at different concentrations [T] of Dps were obtained, the data were plotted with [T] on the horizontal axis and $[T]/\Delta m_A$ on the vertical axis to produce Figure 6(b, d, f). These data points must represent a linear function as seen in Equation (8). The least-squares method was used to obtain the approximate line of the linear function, and the slope and Y-intercept were obtained. Since the slope was $1/\Delta m_{Amax}$, the value of Δm_{Amax} could be determined. Since the Y-intercept is $Kd/\Delta m_{Amax}$, Kd was calculated by multiplying the Y-intercept with Δm_{Amax}.

7 *The monomeric and 12-mer assembly structures of the Dps subunit/linker (SGGG)/aptamer (Y1, Y2, and Y3) predicted by the AlphaFold3.*

We used AlphaFold3 to predict the three-dimensional structure of a Dps subunit with an aptamer attached at its C-terminus via an SGGG linker. The results are shown in Figure S5 where the confidence levels of the predictions are color-coded and predicted Local Distance Difference Test (pLDDT) scores are reported in Table S2.

The native structure of Dps had already been determined and was registered in the Protein Data Bank as 6QHV. A comparison between the predicted structure of the original subunit and the known native Dps structure demonstrated a nearly perfect match Root Mean Square Deviation (RMSD) of the monomer is 0.158 Å (PyMol) (Figure S5(a)). This supports that the structure predicted by AlphaFold3 is in good agreement with the Protein Data Bank model. This confirms the high accuracy of AlphaFold3's structure prediction. Regarding the aptamer structure beyond the linker, the prediction reliability decreases (Figure S5(b), Table S2). The Y1 and Y2 aptamers adopted partially disordered α-helical-

like structures and were positioned parallel to the Dps surface. In contrast, the Y3 aptamer formed a distinct α-helix structure and was slightly open toward the outside relative to the subunit, suggesting that the C-terminus was oriented away from the Dps surface.

We also performed structural prediction for the 12-mer assembly of the Dps subunit/linker (SGGG)/aptamer complex. The results are shown in Figure S5(c)**.** The predicted structure formed a cage-shaped protein, with the protein shell closely matching that of native Dps (RMSD of 12-mer is 0.39 Å (MM-align) Figure S5(c)). The aptamer structures beyond the linker were similar to the monomeric prediction; Y1 and Y2 adopted partially disordered α-helical structures, while Y3 forms a well-defined α-helix. In the 12-mer assembly. All aptamers were oriented nearly parallel to the Dps surface, with Y3Ct exhibiting a slight outward skew.

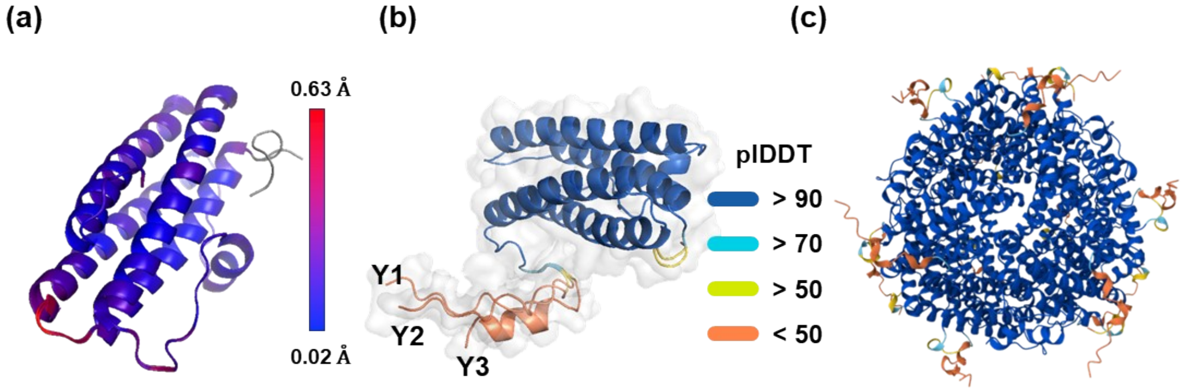

Figure S5. The monomeric and 12-mer assembly structures of the Dps subunit/linker (SGGG)/aptamer (Y1, Y2, and Y3) predicted by AlphaFold3. (a) Superimposition of the AlphaFold3-predicted subunit and X-ray crystallographic structure (6QHV PDB). The positional deviations were indicated by a color bar, ranging from a minimum of 0.02 to a maximum of 0.63 Å. (b) Monomer subunit of the Dps mutant with Y1, Y2, and Y3 superimposed. pLDDT scores are shown in color. (c) 12mer assembly. The threefold axis is shown perpendicular to the paper.

Table S2 Average pLDDT score for each region of mutant Dps.

Region	Residual #	Y1Ct	Y2Ct	Y3Ct
Dps-shell	1-156	94.21	94.18	94.15
Linker	157-160	55.2	54.3	56.1
Aptamer	161-172	34.72	24.31	35.54

8 XPS analysis

Wide- and narrow-scan XPS measurements were performed using a PHI 5000 VersaProbe II spectrometer (ULVAC-PHI Inc., Kanagawa, Japan) equipped with a monochromatic Al Kα source. The acquired spectra of the carbon C1s region were processed with *Igor Pro* (WaveMetrics, Lake Oswego, OR, USA). After background subtraction, the binding energy scale was calibrated by assigning the maximum peak to the sp^2 C–C bulk component (~284.1 eV). Peak positions were determined by interactive fitting with the Voigt function, a convolution of Gaussian and Lorentzian functions. Peak assignments were made with reference to published databases and literature (https://xpsdatabase.net/carbon-spectra-graphene-fresh-peel-from-hopg/, https://www.thermofisher.com/jp/ja/home/materials-science/learning-center/periodic-table/non-metal/carbon.html). The fitted peak data are summarized in Tables S3 and S4.

For the SPE carbon electrode, the spectrum was dominated by the sp^2 C–C peak at ~284.07 eV, with a minor sp^3 contribution at ~285.02 eV and only a very small oxygen-containing peak. In contrast, the QCM carbon electrode exhibited additional oxygen-related components, including C–O–C/C–O–H (~286.32 eV), C=O (~288.2 eV), and π–π* (~290.6 eV). These results suggest that oxygen incorporation occurred during electrode fabrication by vacuum deposition.

Table S3. Data obtained from peak fitting and separation of the C1s region in the XPS spectra of the SPE electrode.

Peak	Reference eV	Position (eV)	Area Ratio	Atomic (%)
sp^2	284.1	284.07	0.88702	78.0
sp^3	284.8	285.02	0.08209	7.22
O-C=O	288.5	288.13	0.03089	2.7

Table S4. Data obtained from peak fitting and separation of the C1s region in the XPS spectra of the QCM electrode.

Peak	Reference eV	Position (eV)	Area Ratio	Atomic (%)
sp^2	284.1	284.29	0.692677	63.2
sp^3	284.8	285.48	0.13082	11.9
O-C=O	288.5	288.2	0.053482	4.9
C-O-C/C-O-H	286	286.32	0.093417	8.5
π-π*	290	290.62	0.029605	2.7
